# Effects of Chrysotile Exposure in Human Bronchial Epithelial Cells: Insights into the Pathogenic Mechanisms of Asbestos-Related Diseases

**DOI:** 10.1289/ehp.1409627

**Published:** 2015-12-18

**Authors:** Giulia Rossana Gulino, Manuela Polimeni, Mauro Prato, Elena Gazzano, Joanna Kopecka, Sebastiano Colombatto, Dario Ghigo, Elisabetta Aldieri

**Affiliations:** 1 Interdepartmental Center for Studies on Asbestos and Other Toxic Particulates “G. Scansetti,” University of Torino, Torino, Italy; 2 Department of Oncology, University of Torino, Torino, Italy; 3 Department of Neurosciences, University of Torino, Torino, Italy

## Abstract

**Background:**

Chrysotile asbestos accounts for > 90% of the asbestos used worldwide, and exposure is associated with asbestosis (asbestos-related fibrosis) and other malignancies; however, the molecular mechanisms involved are not fully understood. A common pathogenic mechanism for these malignancies is represented by epithelial–mesenchymal transition (EMT), through which epithelial cells undergo a morphological transformation to assume a mesenchymal phenotype. In the present work, we propose that chrysotile asbestos induces EMT through a mechanism involving a signaling pathway mediated by tranforming growth factor beta (TGF-β).

**Objectives:**

We investigated the role of chrysotile asbestos in inducing EMT in order to elucidate the molecular mechanisms involved in this event.

**Methods:**

Human bronchial epithelial cells (BEAS-2B) were incubated with 1 μg/cm2 chrysotile asbestos for ≤ 72 hr, and several markers of EMT were investigated. Experiments with specific inhibitors for TGF-β, glycogen synthase kinase–3β (GSK-3β), and Akt were performed to confirm their involvement in asbestos-induced EMT. Real-time polymerase chain reaction (PCR), Western blotting, and gelatin zymography were performed to detect mRNA and protein level changes for these markers.

**Results:**

Chrysotile asbestos activated a TGF-β–mediated signaling pathway, implicating the contributions of Akt, GSK-3β, and SNAIL-1. The activation of this pathway in BEAS-2B cells was associated with a decrease in epithelial markers (E-cadherin and β-catenin) and an increase in mesenchymal markers (α-smooth muscle actin, vimentin, metalloproteinases, and fibronectin).

**Conclusions:**

Our findings suggest that chrysotile asbestos induces EMT, a common event in asbestos-related diseases, at least in part by eliciting the TGF-β–mediated Akt/GSK-3β/SNAIL-1 pathway.

**Citation:**

Gulino GR, Polimeni M, Prato M, Gazzano E, Kopecka J, Colombatto S, Ghigo D, Aldieri E. 2016. Effects of chrysotile exposure in human bronchial epithelial cells: insights into the pathogenic mechanisms of asbestos-related diseases. Environ Health Perspect 124:776–784; http://dx.doi.org/10.1289/ehp.1409627

## Introduction

Asbestos is a nonspecific term that is commonly used to describe any of six types of naturally occurring fibrous silicate minerals that were widely used commercially during the 20th century. Chrysotile asbestos is estimated to account for 90% of the asbestos used worldwide (Qi et al. 2013). Since the beginning of the 20th century, asbestos inhalation has been considered responsible for a number of lung diseases, such as asbestosis (asbestos-induced fibrosis), lung tumors, and malignant mesothelioma (MM) (Kamp 2009). Both fibrosis and epithelial tumors are highly dependent on the ability of epithelial cells to transform into mesenchymal cells through a process called epithelial–mesenchymal transition (EMT).

EMT is both a physiological and pathological process: it has been related to early embryonic development and later organogenesis, as well as to wound healing in fibrotic tissues and to tumor development and metastasis in cancer (Kim et al. 2013; Kim and Cho 2014). During EMT, cell–cell adhesion molecules are inactivated and sometimes destroyed while cell–matrix adhesion increases. Cells undergoing EMT lose epithelial marker proteins, such as the adherent junction proteins E-cadherin and β-catenin and the tight junction protein zonula occludens, and begin to express mesenchymal proteins such as collagen, vimentin, α-smooth muscle actin (α-SMA), and fibronectin (Barrallo-Gimeno and Nieto 2005; Cannito et al. 2010; Moody et al. 2005; Peinado et al. 2004). These events lead to the acquisition of a fibroblast-like and spindle-shaped morphology, and cells acquire the capacity to degrade the basement membrane and migrate through the extracellular matrix to populate different territories during either embryonic development or cancer progression, or to adopt a profibrotic myofibroblast nature (Acloque et al. 2009; Cannito et al. 2010; Kalluri and Neilson 2003; Kalluri and Weinberg 2009; Moustakas and Heldin 2012).

However, the spectrum of changes occurring during EMT may vary significantly depending on the epithelial cell type, the surrounding microenvironment and the type of inducer. Extracellular signals that can trigger EMT include growth factors such as transforming growth factor–β (TGF-β), hepatocyte growth factor, platelet-derived growth factor, fibroblast growth factor, and cytokines such as tumor necrosis factor–α (TNF-α) (Cannito et al. 2010; Chen et al. 2014; Farrell et al. 2014; Moustakas and Heldin 2012).

TGF-β is a multifunctional protein capable of regulating cell growth and differentiation as well as stimulating the production of extracellular matrix (Fine and Goldstein 1987). The different roles of TGF-β have been widely explored: TGF-β exerts its biological activity by regulating growth, differentiation, and epithelial transformation in the multistep processes of carcinogenesis, wound healing, and embryogenesis (Bhowmick et al. 2001; Perdue and Brody 1994).

Many studies have investigated the effects of asbestos. Asbestos induces lung fibrosis via increased secretion of TGF-β (Manning et al. 2002), particularly in idiopathic pulmonary fibrosis where TGF-β has been localized in association with bronchiolar epithelial cells and their extracellular matrix (Liu and Brody 2001; Pociask et al. 2004). Casarsa et al. (2011) stressed the importance of EMT markers in MM prognosis. Qi et al. (2013) compared the toxicity of two different kinds of asbestos: chrysotile and crocidolite [an amphibole asbestos that is often considered the most oncogenic type of asbestos (Gibbs and Berry 2008)]. In their work, Qi et al. suggested that continuous exposure to crocidolite and chrysotile could cause EMT of human mesothelial cells via High Mobility Group Box 1 (HMGB1) and TNF-α signaling. In particular, the authors found that repeated exposure to chrysotile and crocidolite led to similar molecular changes and to similar amounts of HMGB1 secretion *in vitro* and *in vivo*, with differences in inducing MM-related biological alterations according to their biopersistence (Qi et al. 2013). For these reasons, interest in the role of asbestos as an inducer of EMT has recently increased.

Given the strong association of chrysotile exposure with TGF-β activation (Murthy et al. 2015; Pociask et al. 2004), which is in turn associated with EMT induction, we investigated the role of chrysotile in inducing EMT via TGF-β in a human bronchial epithelial cellular model (BEAS-2B) to increase knowledge of the molecular bases of asbestos-related lung diseases.

## Materials and Methods

### Asbestos Samples

UICC (Union International Contre le Cancer) chrysotile and UICC crocidolite were sonicated (100 W, 30 sec, Labsonic Sonicator; Sartorius Stedim Biotech S.A.) before incubation with cell cultures to dissociate fiber bundles and to improve their suspension in the culture medium.

### Cell Cultures

BEAS-2B cells (immortalized human bronchial epithelial cells) were obtained from American Type Culture Collection (ATCC). They were cultured in Roswell Park Memorial Institute (RPMI) 1640 medium (Gibco) supplemented with 10% fetal bovine serum (FBS) and 1% penicillin/streptomycin. Human bronchial epithelial cells (NuLi-1) were a generous gift from C. Voena (Department of Molecular Biotechnology and Health Sciences, University of Torino). NuLi-1 cells were cultured in serum-free medium (Bronchial Epithelial Cell Growth Medium; Lonza) in Petri dishes that had been precoated with a 60 μg/mL solution of human placental collagen type IV at least 18 hr in advance, then air-dried and rinsed two to three times with phosphate-buffered saline (PBS).

Human lung adenocarcinoma alveolar epithelial cells (A549) were provided by Istituto Zooprofilattico Sperimentale “Bruno Ubertini” (Brescia, Italy). The cells were cultured in Ham’s F12 medium (Gibco) supplemented with 10% FBS and 1% penicillin/streptomycin.

All cell cultures were kept in a humidified incubator at 37°C in a 5% CO_2_ atmosphere.

### Experimental Conditions

Dose- and time-dependence experiments were performed to determine the appropriate concentration and time, respectively, for incubating BEAS-2B cells with chrysotile or crocidolite (data not shown). As a consequence of these preliminary results, we chose to seed 3.5 × 10^5^ or 1.5 × 10^5^ BEAS-2B cells in 100-mm–diameter Petri dishes and incubate them for ≤ 72 hr or for 7 days, respectively, in the absence or presence of 1 μg/cm^2^ chrysotile asbestos or 5 μg/cm^2^ crocidolite. In the same manner, after preliminary experiments, 1.5 × 10^5^ NuLi-1 cells were seeded in 60-mm–diameter Petri dishes and incubated for 96 hr in the absence or presence of 1 μg/cm^2^ chrysotile. In addition, 1.5 × 10^5^ A549 cells were seeded in 100-mm–diameter Petri dishes and then incubated for 96 hr with 5 μg/cm^2^ chrysotile.

The protein content in the cells was detected using a bicinchoninic acid assay (BCA) kit (Sigma Chemical Co.). The plasticware for cell culture was provided by Falcon (Corning Incorporated). Ultrapure water (Millipore) was used for all experiments.

### Cell Morphology

At the end of the incubation period, cells were observed using a light microscope, and images were obtained with the Leica Application Suite program (Leica Microsystems).

### Measurement of Reactive Oxygen Species (ROS)

BEAS-2B cells were incubated for 30 min or 1, 3 or 6 hr in the absence or presence of 1 μg/cm^2^ of chrysotile; then, cells were incubated for 30 min with 10 μM 2´,7´-dichlorodihydrofluorescein diacetate (DCFH-DA). DCFH-DA is a cell-permeable probe that is cleaved intracellularly by nonspecific esterases to form DCFH, which is further oxidized by ROS to form the fluorescent compound dichlorofluorescein (DCF) in a 1:1 stoichiometry (Bass et al. 1983). After incubation with DCFH-DA, the cells were washed twice with PBS to remove excess probe, and DCF fluorescence was determined using a Synergy HT microplate reader (BioTek Instruments) at an excitation wavelength of 504 nm and an emission wavelength of 529 nm. The fluorescence value was normalized to protein concentration and expressed as units of arbitrary fluorescence.

### Western Blot Analysis

Cytosolic and nuclear extracts were obtained using an Active Motif nuclear extraction kit (Active Motif) according to the manufacturer’s instructions.

Cytosolic and nuclear extracts were separated by sodium dodecyl sulfate–​polyacrylamide gel electrophoresis (SDS-PAGE), transferred to polyvinylidene difluoride (PVDF) membrane sheets (Immobilon-P, Millipore) and probed with the required antibody diluted in 0.1% PBS-Tween with 5% nonfat dry milk. After 1 hr of incubation, the membranes were washed with 0.1% PBS-Tween and then were incubated for 1 hr with peroxidase-conjugated sheep anti-mouse or sheep anti-rabbit IgG antibody (Amersham International) diluted 1:3,000 in 0.1% PBS-Tween with 5% nonfat dry milk. The membranes were washed again with 0.1% PBS-Tween, and proteins were detected by enhanced chemiluminescence (Perkin Elmer).

Anti-E-cadherin, β-catenin, tubulin, SNAIL-1, and TATA-binding protein (TBP) antibodies were all provided by Santa Cruz Biotechnology, Inc. Tubulin and TBP were used as loading controls for the cytosol and the nucleus, respectively. The anti-vimentin antibody was provided by Sigma Chemical Co. The anti-α-SMA antibody was from GeneTex. The anti-Smad2 and p-Smad2 antibodies were from Abcam.

### Specific Inhibitors

The neutralizing anti–TGF-β antibody was purchased from Abcam and was used at a concentration of 5 μg/mL; the GSK-3β inhibitor SB 216763 and the Akt 1/2 kinase inhibitor were from Sigma and were both used at a concentration of 5 μM.

### Quantitative Real-Time PCR (qRT-PCR)

Total RNA was obtained by the guanidinium thiocyanate–phenol–chloroform method (Chomczynski and Sacchi 1987), using RiboZol RNA Extraction Reagents (Amresco)according to the manufacturer’s instructions. Total RNA (0.2 μg) was reverse-transcribed into cDNA using an iScript cDNA Synthesis Kit (Bio-Rad Laboratories AG) according to the manufacturer’s instructions. qRT-PCR was performed using IQ™ SYBR Green Supermix (Bio-Rad) according to the manufacturer’s instructions. PCR amplification was performed as follows: 1 cycle of denaturation at 94°C for 3 min, 45 cycles of denaturation at 94°C for 30 sec, annealing for 30 sec, and synthesis at 72°C for 30 sec.

The relative expression of each target gene was determined by comparing each PCR gene product with the S14 ribosomal subunit product using the Gene Expression Macro (http://www3.bio-rad.com/LifeScience/jobs/2004/04-0684/genex.xls; Bio-Rad).

### Quantification of TGF-β Secretion by ELISA

After incubating BEAS-2B cells in the absence or presence of chrysotile, the extracellular medium was collected and centrifuged at 4°C at 13,000 × *g* for 30 min. To determine the concentration of TGF-β in the supernatant, ELISA was performed according to the manufacturer’s instructions (Invitrogen Corporation). Absorbance was measured at 450 nm with a Synergy HT microplate reader. The amount of cytokine was determined using a standard curve and was corrected for the content of cell protein. The results were expressed as pg/mg of intracellular protein.

### Gelatin Zymography

Because FBS contains matrix metalloproteinases (MMPs), cells were cultured in 1% serum medium only. Afterwards, the supernatant was collected, supplemented with Laemmli sample buffer, and subjected to 10% SDS-PAGE with 1 mg/mL gelatin under nondenaturing and nonreducing conditions as previously described (Giribaldi et al. 2011).

### Statistical Analysis

Where appropriate, data in figures are reported as the mean ± SEM. The results were analyzed by one-way analysis of variance (ANOVA) and Tukey’s test (SPSS 11.0 for Windows, SPSS Inc.); *p* < 0.05 was considered significant.

## Results

### Effect of Chrysotile and Crocidolite on EMT in BEAS-2B Cells

**Figure 1:**
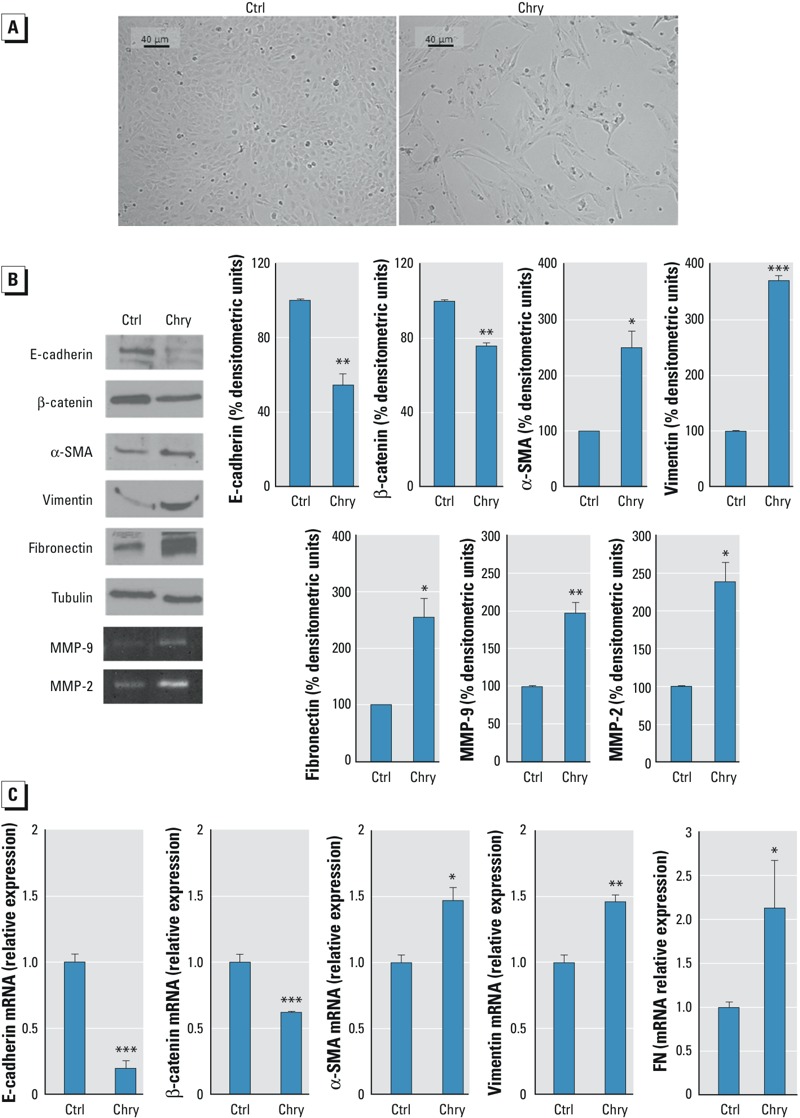
Effects of chrysotile asbestos on cell morphology and epithelial–mesenchymal transition (EMT) marker protein levels in BEAS-2B cells. BEAS-2B cells were cultured for 72 hr without (Ctrl) or with 1 μg/cm^2^ chrysotile (Chry). (*A*) Representative microscopy images are shown (10**×**; scale bar = 40 μm). (*B*) Expression of epithelial (E-cadherin, β-catenin) and mesenchymal (α-SMA, vimentin, fibronectin) markers checked by Western blotting and evaluation of MMP-2 and MMP-9 activity by zymography. Tubulin was used as a loading control. The image is representative of three independent experiments that produced similar results. Densitometry data are presented as the percent decrease or increase in the protein levels**versus the**respective control. Significance versus the respective control: *p < 0.005; **p < 0.001; ***p < 0.0001. (*C*) Relative gene expression of E-cadherin, β-catenin, α-SMA, vimentin, fibronectin (FN) evaluated by quantitative real-time polymerase chain reaction (qRT-PCR). Data are expressed in units of relative mRNA expression compared with control cells (*n* = 3). Significance versus the respective control: *p < 0.02; **p < 0.001; ***p < 0.0001.

After 72 hr of incubation with 1 μg/cm^2^ chrysotile, BEAS-2B cells acquired a spindle-shaped fibroblast-like morphology similar to that observed after stimulation with TGF-β, which was previously documented by Doerner et al. (Doerner and Zuraw 2009) and is typical of EMT (Figure 1A). After 72 hr of incubation with chrysotile, BEAS-2B cells showed significantly decreased levels of the proteins E-cadherin and β-catenin compared with untreated cells. In parallel, markers commonly associated with a mesenchymal phenotype, such as α-SMA, vimentin, and fibronectin, were significantly increased (Figure 1B). All of the markers were also analyzed with qRT-PCR to investigate changes in gene expression; the results of these experiments were similar to those observed for the Western blot experiments (Figure 1C). Because MMPs play a fundamental role in extracellular matrix remodeling and are markers of EMT (Cannito et al. 2010; Kessenbrock et al. 2010), we investigated the secretion and activity of two MMPs. Cells exposed to chrysotile secreted more MMP-2 and MMP-9 than untreated cells (Figure 1B).

**Figure 2:**
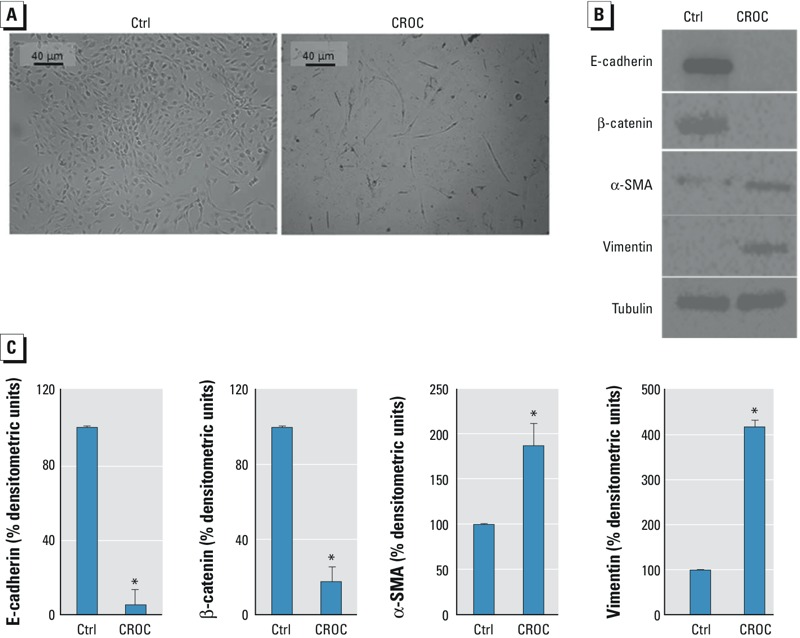
Effect of crocidolite exposure on cell morphology and alterations in proteins involved in epithelial–mesenchymal transition (EMT) in BEAS-2B cells. BEAS-2B cells were cultured for 7 days without (Ctrl) or with 5 μg/cm^2^ crocidolite (CROC). (*A*) Representative microscopy images are shown (10×; scale bar = 40 μm). (*B*,*C*) Expression of epithelial (E-cadherin, β-catenin) and mesenchymal (α-SMA, vimentin) markers checked by Western blotting. Tubulin was used as a loading control. The image is representative of three independent experiments that produced similar results. Densitometry data are presented as the percent decrease or percent increase in the protein levels versus the respective control. Significance versus the respective control: *p < 0.0001.

BEAS-2B cells were also incubated with 5 μg/cm^2^ crocidolite for 7 days. After incubation, cells observed by optical microscopy had lost their organization and assumed a fibroblast-like appearance with pointed ends and elongated protrusions (Figure 2A). Expression of E-cadherin and β-catenin was significantly decreased in cells exposed to crocidolite, and the mesenchymal proteins (α-SMA and vimentin) were significantly over-expressed under the same experimental conditions compared with untreated cells (Figure 2B,C).

### Effects of Chrysotile on EMT in NuLi and A549 Cells

We performed experiments to confirm the effects of chrysotile on a human bronchial epithelial cell line (NuLi-1) and on a human lung adenocarcinoma cell line (A549). After incubation with chrysotile, NuLi-1 and A549 cells were observed by optical microscopy; then, the expression of epithelial and mesenchymal markers was evaluated by Western blotting. Asbestos-treated cells had lost their organization in compact islets and had taken on a tapered and spindle-like shape with pointed ends and elongated protrusions, assuming a fibroblast-like appearance, whereas the control cells showed typical epithelial morphology. The Western blotting data revealed that the levels of epithelial markers (E-cadherin and β-catenin) significantly decreased in cells treated with chrysotile, whereas compared with untreated cells, mesenchymal proteins (α-SMA and vimentin) were significantly over-expressed under the same experimental conditions (see Figures S1–S2).

### Effects of Chrysotile on TGF-β Secretion and ROS Production in BEAS-2B Cells

**Figure 3:**
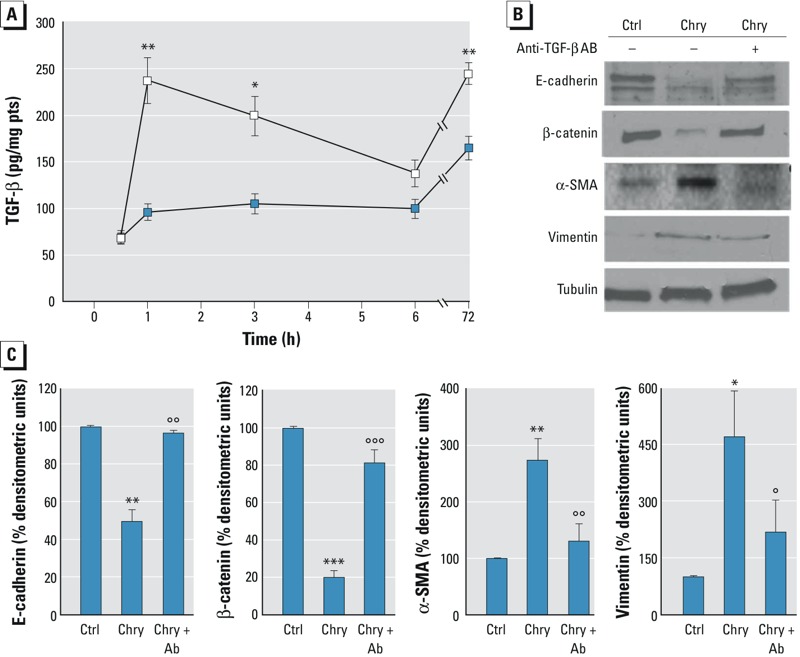
TGF-β secretion and neutralizing TGF-β antibody effect in BEAS-2B cells exposed to chrysotile. (*A*) BEAS-2B cells were incubated in the absence (blue squares) or presence (white squares) of 1 μg/cm^2^ chrysotile for 30 min and 1, 3, 6 and 72 hr. Afterwards, the supernatants were collected, and TGF-β levels were detected using an ELISA kit. Data are shown as the mean ± SEM (*n* = 3). TGF-β levels are reported as picograms per milligram of intracellular protein. Significance versus the respective control: *p < 0.05; **p < 0.02. (*B*,*C*) BEAS-2B cells were incubated without (Ctrl) or with 1 μg/cm^2^ chrysotile (Chry) or with chrysotile and 5 ng/mL of neutralizing anti–TGF-β antibody for 72 hr (Chry + Ab). The expression of epithelial (E-cadherin, β-catenin) and mesenchymal (α-SMA, vimentin) markers was determined by Western blotting. Tubulin was used as a loading control. The image is representative of three independent experiments. Densitometry data are presented as the percent decrease or increase versus control cells. Significance versus the respective control: *p < 0.005; **p < 0.001; ***p < 0.0001. Significance versus chrysotile: °*p *< 0.005; °°*p *< 0.001; °°°*p *< 0.0001. No significant differences were detected for cells coincubated with chrysotile and TGF-β antibody compared with control cells.

Because the above-mentioned markers (E-cadherin, β-catenin, α-SMA, vimentin, fibronectin and MMPs) are strongly associated with TGF-β–induced EMT (Cannito et al. 2010; Kessenbrock et al. 2010; Kondo et al. 2004), we investigated the possible involvement of TGF-β in the effects observed in our cellular model. The levels of TGF-β were assessed in the supernatants of BEAS-2B cells that had been incubated with 1 μg/cm^2^ chrysotile for 30 min and for 1, 3, and 6 hr. TGF-β levels significantly increased after 1 hr of incubation, then decreased to a level that was not significantly different from controls at 6 hr (Figure 3A). When incubation was extended to 72 hr, TGF-β levels were increased in both treated and untreated cells but were significantly higher in treated cells (244.75 ± 11.75 pg/mg protein) than in control cells (164.77 ± 12.62 pg/mg protein) (*p* < 0.05 for the mean ± SEM from three independent experiments; see Figure 3A).

Chrysotile exposure has already been associated with the increased activation of TGF-β (Liu and Brody 2001; Manning et al. 2002), and ROS generated by the iron contained in asbestos can mediate the biological activity of TGF-β (Pociask et al. 2004). As shown in Figure S3, we detected significant production of ROS after 30 min incubation of BEAS-2B cells with chrysotile: ROS remained high until 1 hr of incubation and then slowly decreased to control levels after 6 hr. These results are consistent with a mechanism whereby ROS cause the early activation of TGF-β.

A neutralizing anti–TGF-β antibody was used to confirm whether TGF-β was a mediator of the changes in E-cadherin, β-catenin, α-SMA and vimentin protein levels. E-cadherin and β-catenin levels were significantly lower and α-SMA and vimentin levels were significantly higher in cells treated with chrysotile than in untreated cells. The coincubation of BEAS-2B cells with both chrysotile and the TGF-β–blocking antibody partially restored the protein levels (Figure 3B,C). No changes in protein expression were observed in BEAS-2B cells incubated with neutralizing anti–TGF-β antibody alone (see Figure S4).

### Effects of Chrysotile Exposure on the Smad2-Dependent and Independent Pathways

TGF-β is responsible for the activation of a canonical pathway mediated by the intracellular effectors Smad proteins (Xie et al. 2014). To investigate whether any change in the levels of Smad2 and phospho-Smad2 (p-Smad2) proteins occurred in our cellular model, we performed time-dependence experiments in which BEAS-2B cells were incubated with 1 μg/cm^2^ chrysotile for up to 6 hr and evaluated the levels of Smad2 and p-Smad2 in the cytosolic and nuclear fractions at each time point by Western blotting. As shown in Figure S5 (panel A), after 30 min of incubation with chrysotile, the level of p-Smad2 decreased in the cytoplasm and contemporaneously increased in the nuclei. The increase was evident and significant after 30 min and was significant up to 6 hr of incubation (see Figure S5, panel B).

To further elucidate the mechanisms leading to the down-regulation of E-cadherin, which is typical of EMT (Lamouille et al. 2014), we investigated the involvement of the transcription factor SNAIL-1. The subcellular localization and degradation of SNAIL-1 are highly dependent on glycogen synthase kinase–3β (GSK-3β) (Zhou et al. 2004). Normally, GSK-3β induces phosphorylation of nuclear SNAIL-1, mediating its nuclear export and subsequent cytosolic degradation; nevertheless, the phosphorylation and functional inactivation of GSK-3β is crucial to stabilize SNAIL-1 in the nucleus, where it down-regulates the E-cadherin gene (*CDH1*) (Zhou et al. 2004).

**Figure 4:**
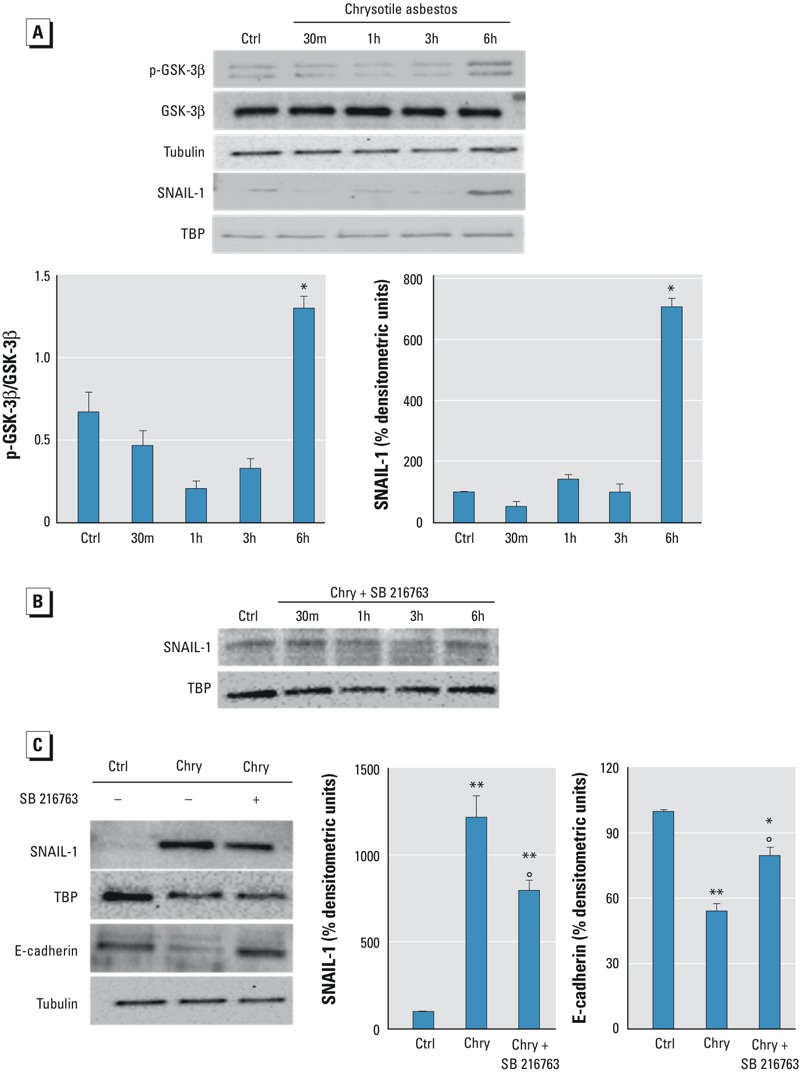
Evaluation of the role of the GSK-3β/SNAIL-1 pathway in E-cadherin gene modulation in BEAS-2B cells. The images are representative of three independent experiments that produced similar results. (*A*) BEAS-2B cells were incubated in the absence (Ctrl, 6 hr) or presence of 1 μg/cm^2^ chrysotile for 30 min and 1, 3 and 6 hr (top panel). The expression of phosphorylated GSK-3β (p-GSK-3β) and the accumulation of SNAIL-1 in the nuclei of BEAS-2B cells were examined by Western blotting. p-GSK-3β to GSK-3β ratio values are shown (bottom panel, left). Densitometry data concerning SNAIL-1 accumulation in the nuclei are presented as the percent decrease or increase in the protein levels versus control (Ctrl, 6 hr) (bottom panel, right). Significance versus control (Ctrl, 6 hr): *p < 0.0001. (*B*) BEAS-2B cells were treated without (Ctrl, 6 hr) or with chrysotile (Chry, 1 μg/cm^2^) together with the specific GSK-3β inhibitor SB216763 (5 μM) for 30 min and 1, 3 and 6 hr. Tubulin and TATA-binding protein (TBP) were used as loading controls for the cytosol and the nucleus, respectively. (*C*) BEAS-2B cells were treated for 72 hr without (Ctrl) or with chrysotile (1 μg/cm^2^) in the absence (Chry) or presence (Chry + SB) of SB216763 (5 μM) (left panel). Experiments were performed in triplicate, and densitometry data are presented as the percent decrease or increase in the protein levels vesus the respective control (right panel). Significance versus the respective control: *p < 0.005; **p < 0.0001. Significance versus chrysotile: °*p *< 0.001.

In our experimental model, to assess the roles of both GSK-3β and SNAIL-1, we performed time-dependence experiments. A 6-hr exposure to chrysotile resulted in GSK-3β phosphorylation and SNAIL-1 accumulation in the nucleus (Figure 4A). To confirm the role of GSK-3β in allowing the nuclear stabilization of SNAIL-1, we performed time-dependence experiments in which the coincubation of BEAS-2B cells with chrysotile and SB216763, a specific inhibitor of GSK-3β (Coghlan et al. 2000), prevented the increase of SNAIL-1 in the nucleus (Figure 4B). Moreover, immunoblot analysis of BEAS-2B cells incubated with both chrysotile and SB216763 for 72 hr showed that although chrysotile increased SNAIL-1 levels in the nucleus and decreased E-cadherin levels in the cytosol, coincubation with the GSK-3β inhibitor partially reversed both the nuclear increase of SNAIL-1 and the cytosolic decrease of E-cadherin induced by chrysotile, resulting in a statistically significant difference compared with both chrysotile-incubated and control levels (Figure 4C).

**Figure 5:**
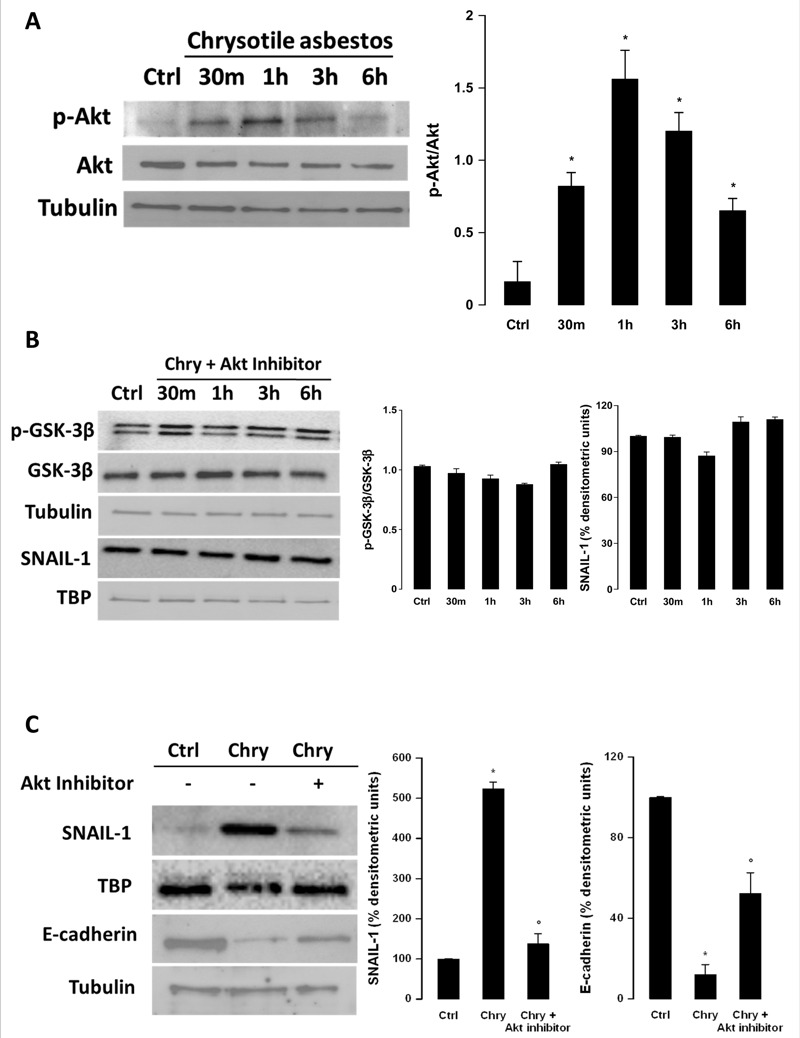
Evaluation of Akt involvement in GSK-3β regulation in BEAS-2B cells. The images are representative of three independent experiments that produced similar results. (*A*) BEAS-2B cells were incubated in the absence (Ctrl, 6 hr) or presence of chrysotile (1 μg/cm^2^) from 30 min up to 6 hr (left panel). Differences in the p-Akt (phosphorylated Akt) to Akt ratio are shown in the right panel. Significance versus control: *p < 0.0001. (*B*) BEAS-2B cells were treated without (Ctrl) or with chrysotile (Chry, 1 μg/cm^2^) together with the Akt inhibitor (5 μM) from 30 min up to 6 hr. p-GSK-3β = phosphorylated GSK-3β. Tubulin and TATA-binding protein (TBP) were used as loading controls. Differences in the p-GSK-3β to GSK-3β ratio and densitometry data as the percent increase or decrease in SNAIL-1 levels are presented in the right panel. (*C*) BEAS-2B cells were treated for 72 hr without (Ctrl) or with chrysotile alone (Chry, 1 μg/cm^2^) or together with 5 μM Akt inhibitor (left panel). Tubulin and TBP were used as loading controls. Densitometry data are presented as the percent decrease or increase in the protein levels versus the respective control (right panel). With regard to the SNAIL-1 densitometry data, no significant differences were detected for cells coincubated with chrysotile and Akt inhibitor compared with control cells. Significance versus the respective control: *p < 0.0001. Significance versus chrysotile: °*p *< 0.0001.

GSK-3β is a ubiquitously expressed serine–threonine kinase that is involved in many different signaling pathways (Pap and Cooper 1998). Among these pathways, we hypothesized that GSK-3β phosphorylation could result from activation of the Smad-independent pathway of TGF-β signaling that would involve Akt as an upstream kinase for GSK-3β. The ratio of phosphorylated to nonphosphorylated Akt (p-Akt/Akt) was increased after incubating BEAS-2B cells with chrysotile for 30 min to 3 hr but was not significantly different from control levels at 6 hr (Figure 5A). The involvement of Akt in this pathway was confirmed by incubating the BEAS-2B cells with chrysotile together with the specific Akt 1/2 kinase inhibitor. The results indicated that the Akt inhibitor kept both the phosphorylation levels of GSK-3β and the nuclear stabilization of SNAIL-1 unaltered compared with the control levels (Figure 5B). Furthermore, although chrysotile increased SNAIL-1 in the nucleus and decreased E-cadherin in the cytosol after incubation for 72 hr, co-incubation with the specific Akt inhibitor partially restored the levels of both proteins (Figure 5C).

## Discussion

The carcinogenicity of asbestos is well documented (IARC 2012), and consequently, many countries throughout the world have banned all forms of asbestos from commercial use. However, ≥ 125 million workers worldwide continue to be exposed to asbestos fibers, among whom 1.2 million are in the European Union (IARC 2012). Furthermore, asbestos continues to be used in developing countries, and exposure to asbestos in nonoccupational settings and in the general environment remains a serious health concern (Birnbaum et al. 2010). Moreover, it is also problematic that countries with growing economies, such as China, India, and Russia, are the largest consumers of asbestos (LaDou 2004). Furthermore, asbestos is widespread in the environment, where fibers occur in natural deposits and as contaminants of other minerals. Fibers can be released from the weathering of asbestos-bearing rocks as well as through anthropogenic activities (IARC 2012).

Because all forms of asbestos (chrysotile, crocidolite, amosite, tremolite, actinolite, and anthophyllite) have been associated with an increased risk of lung cancer and mesothelioma (IARC 2012), it is estimated that asbestos-related pathologies will increase in the coming years, both because of the long latency period between asbestos exposure and the onset of any related pathologies and because of the absence of restrictions in the use of asbestos in many countries throughout the world (Robinson 2012).

The results of the present study represent the first steps towards understanding some of the molecular mechanisms involved in EMT, a common event in all asbestos-related pathologies (Batra and Antony 2015; Guarino et al. 2009; Qi et al. 2013; Tamminen et al. 2012). Elucidating the molecular mechanisms underlying such a complex scenario can be of great help in discovering crucial alterations in the cellular microenvironment that could eventually be used as biomarkers of risk for asbestos-associated pathologies.

Asbestos exposure has been associated with the development of fibrosis, lung tumors, and MM (Hodgson and Darnton 2000). EMT plays a fundamental role in all of these pathological conditions (Kalluri and Weinberg 2009). In many cellular models, TGF-β is known to be one of the main EMT inducers, and it has already been reported to mediate asbestos-induced fibrosis (Pociask et al. 2004; Sullivan et al. 2008). However, despite this important evidence, we are not aware of a clear mechanism connecting chrysotile and EMT induction in the current literature.

In the present study, we have provided information about one of the possible mechanisms by which chrysotile triggers EMT in a lung epithelial cellular model. ROS play an important role in asbestos-mediated regulation of different signal transduction pathways (Kamp 2009; Mossman and Churg 1998; Shukla et al. 2003), and they can work as intracellular effectors that are partially responsible for the molecular response to oxidative stress. Nevertheless, few studies have focused on the ability of asbestos to induce EMT through ROS-mediated mechanisms. In 2012, Tamminen et al. carried out a study on A549 cells using crocidolite (Tamminen et al. 2012) and suggested that crocidolite-mediated ROS production could induce EMT through a mechanism involving the MAPK/ERK signaling pathway but was not dependent on the activation of TGF-β signaling. In another study, Sullivan et al. (2008) proposed a model whereby asbestos elicited TNF-α expression. According to their findings, TNF-α controlled fibrogenesis by regulating TGF-β expression, and asbestos-induced ROS triggered lung fibrosis by activating latent TGF-β.

Moreover, Kim et al. analyzed ROS-induced EMT in human malignant mesothelioma (HMM) cells, and based on their findings, they suggested that oxidative stress induced by H_2_O_2_ may play a critical role in HMM carcinogenesis, which would involve TGF-β, hypoxia inducible factor–1α, and some genes related to the capacity of cells to preserve their undifferentiated phenotype (stemness genes) (Kim et al. 2013; Potten and Loeffler 1990). Qi et al. (2013) observed that chrysotile has limited transforming potential *in vitro* compared with crocidolite and stated that the morphological and molecular alterations induced by both crocidolite and chrysotile are suggestive of EMT in human mesothelial cells. They also hypothesized that chrysotile induces transient effects because its biopersistence is different from that of crocidolite (Qi et al. 2013). Starting from these data, we investigated whether chrysotile could induce EMT and which mechanism could be involved in such a transformation.

In epithelial cells, E-cadherin is known to mediate cell–cell tight junctions that are stabilized by β-catenin (Stappert and Kemler 1994); the loss or downregulation of E-cadherin during EMT results in destabilization of the cadherin/catenin complex and disassembly of adherens junctions (Pectasides et al. 2014). In our cellular model, the levels of E-cadherin and β-catenin significantly decreased after chrysotile exposure, confirming the loss of epithelial characteristics of BEAS-2B. At the same time, mesenchymal proteins such as α-SMA and vimentin increased, suggesting the onset of cytoskeleton-related rearrangements typical of EMT (Lamouille et al. 2014). Furthermore, the increased deposition of fibronectin and the extrusion of both MMP-2 and MMP-9 in cells exposed to chrysotile suggest that important changes occurred in the surrounding microenvironment to make the extracellular matrix more suitable to be degraded and invaded (Peinado et al. 2003). We confirmed similar results in other cell lines (NuLi-1 and A549 cells), and a comparable cellular transformation was also observed when we exposed BEAS-2B cells to crocidolite, one of the most carcinogenic asbestos fibers (Takeuchi and Morimoto 1994). Indeed, cells lost their organization and assumed a fibroblast-like appearance, confirming the loss of epithelial morphology and the gain of a mesenchymal phenotype.

As previously reported, chrysotile is known to induce TGF-β activation in a cell-free model (Pociask et al. 2004). In our cellular model, the ELISA assay performed on the cell supernatants confirmed a significant increase of TGF-β levels after 1 hr of incubation with chrysotile, which diminished but was still significantly increased after 3 hr, and which returned near to baseline levels at 6 hr. Because BEAS-2B cells surviving chrysotile exposure for 72 hr developed a morphology suggestive of EMT (spindle-shaped morphology and alterations in epithelial and mesenchymal marker proteins) (Cannito et al. 2010), we also measured the TGF-β levels after 72 hr incubation with chrysotile, that is, after a length of time corresponding to the one we used to detect EMT markers. The levels of TGF-β were significantly higher in treated cells than in control cells.

**Figure 6:**
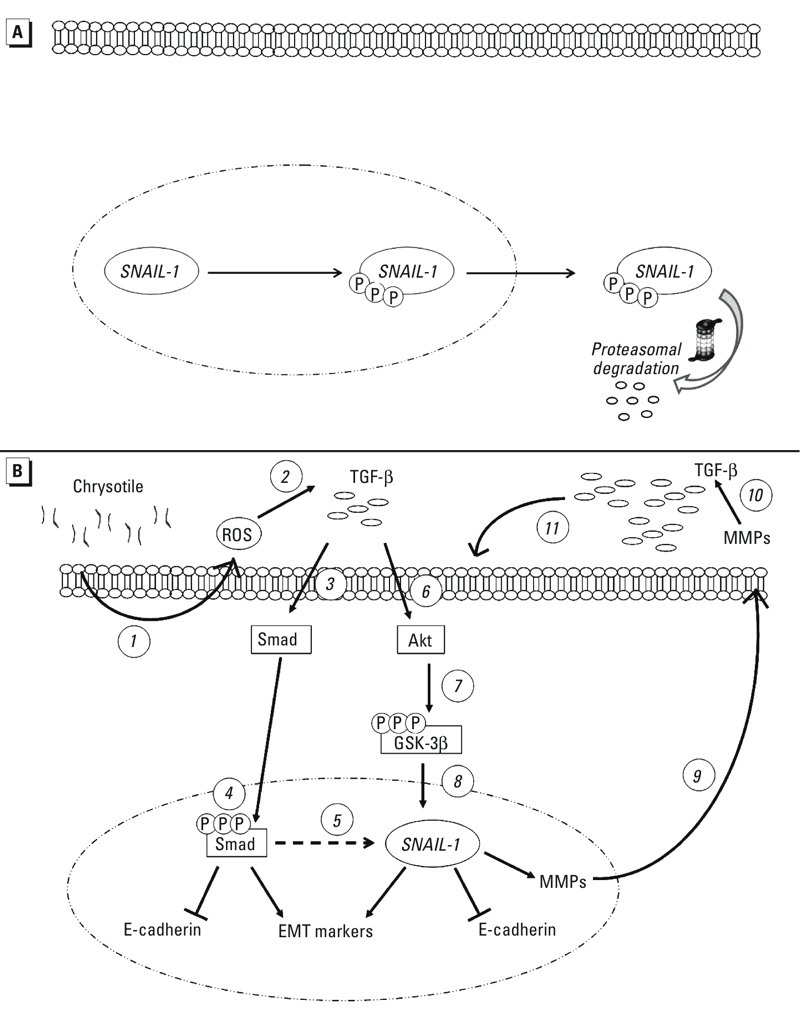
Selected mechanisms potentially involved in TGF-β–mediated epithelial–mesenchymal transition (EMT). (*A*) Under normal conditions, GSK-3β-mediated phosphorylation of nuclear SNAIL-1 allows its nuclear export and subsequent cytosolic degradation (Zhou et al. 2004). (*B*) In our study on BEAS-2B cells, chrysotile exposure induced early production of ROS (*1*), which we hypothesize subsequently activates the latent form of TGF-β (*2*). Upon release, TGF-β is stabilized and directly presented to its receptors, which then associate and activate a variety of signaling pathways. Both Smad-mediated (*3*) and non–Smad-mediated (*6*) pathways are involved (Peinado et al. 2003; Xie et al. 2014). Increased levels of TGF-β result in the activation of the Smad-mediated pathway, suppression of epithelial genes (e.g., E-cadherin), and induction of EMT mesenchymal markers (*4*) through activation/induction of and coassociation with a variety of transcription factors (including SNAIL-1) (*5*). We hypothesize that the non–Smad-mediated pathway leads to Akt activation (*6*) and GSK-3β inactivation (*7*), and induction of SNAIL-1 nuclear stabilization and genes targeting promotion (*8*), thus contributing to EMT. In addition, we hypothesize that MMPs secreted by cells (*9*) may promote the late activation of TGF-β (*10*), resulting in potentially continuous autocrine cell stimulation that may further reinforce signaling for EMT promotion (*11*).

Chrysotile exposure and ROS generated by the iron contained in asbestos have already been associated with the increased activation of TGF-β (Liu and Brody 2001; Manning et al. 2002; Pociask et al. 2004). ROS can activate the latent form of TGF-β (Pociask et al. 2004); therefore, the early ROS production we observed in BEAS-2B cells incubated with chrysotile may be responsible for the first spike of TGF-β. TGF-β can also be activated by many different proteases, including MMPs (Yu and Stamenkovic 2000), the secretion and activity of which were increased after chrysotile exposure. Therefore, it is reasonable to think that because the bioavailability of active TGF-β ligand is greatly dependent on its activation, the alterations caused by chrysotile in the microenvironment of our cellular model (increased production of ROS and secretion of MMPs) could be responsible for the late activation of TGF-β that we detected. Thus, we hypothesize that the initial increase in ROS following chrysotile exposure activates latent TGF-β, which in turn activates the Akt/GSK-3β/SNAIL-1 signaling pathway, and the increase in TGF-β secretion 72 hr after the initial exposure may result from continuous autocrine cell stimulation and may reinforce signaling mechanisms involved in EMT promotion, as summarized in Figure 6. We confirmed the involvement of TGF-β in our cellular model by using an anti–TGF-β neutralizing antibody. The blocking antibody partially restored the levels of epithelial and mesenchymal markers induced by chrysotile alone, thus suggesting an important role for TGF-β in the reported EMT-related morphological alterations.

Moreover, it is well known that TGF-β is responsible for the activation of a canonical pathway mediated by the intracellular effectors, Smad proteins (Xie et al. 2014). According to the literature, once TGF-β binds its receptor TGF-βRI, the recruitment of phosphorylated Smad2 and Smad3 occurs; then, the phosphorylated Smad2/3 binds Smad4 to form a Smad heterocomplex to mediate signal transduction. The TGF-β/Smad signaling pathway can induce or enhance EMT, invasion, and metastasis (Xie et al. 2014). Our findings support the involvement of the canonical TGF-β-mediated Smad-dependent pathway. However, to increase our knowledge about the molecular mechanisms involved in these TGF-β– and chrysotile-mediated modifications, we investigated the noncanonical Akt/GSK-3β/SNAIL-1 pathway. For this reason, we referred to previous works in the literature reporting that TGF-β was responsible for the up-regulation of the transcription factor SNAIL-1, which was in turn implicated in the down-regulation of *CDH1* (Peinado et al. 2003; Zhou et al. 2004). SNAIL-1 persistence in the nucleus is required for the down-regulation of *CDH1* (Zhou et al. 2004). We observed a time-dependent stabilization of SNAIL-1 in the nucleus of BEAS-2B cells incubated with chrysotile. This event may be a result of the phosphorylation of GSK-3β that is a crucial, and often central, component of many cellular functions, contributing to the regulation of apoptosis, cell cycle, cell polarity and migration, gene expression, and many other functions, including the response to inflammatory stimuli (Jope et al. 2007).

We detected increased phosphorylation of GSK-3β accompanied by a simultaneous increase of SNAIL-1 in the nucleus. The involvement of GSK-3β in the regulation of SNAIL-1 was confirmed by coincubating SB216763, a specific inhibitor of GSK-3β, in the presence of this compound. SNAIL-1 increase in the nucleus was partially prevented and E-cadherin was increased, but not completely restored.

GSK-3β phosphorylation is reported to be involved in many different intracellular pathways, including the Wnt (Wu et al. 2012), lipid kinase phosphatidylinositol-4,5-bisphosphate 3-kinase (PI3K)/Akt (Liang and Slingerland 2003), and ERK1/2 MAPK pathways (Kim et al. 2003). We investigated whether Akt could be involved as an upstream kinase for GSK-3β. After exposure to chrysotile, the p-Akt/Akt ratio increased in a time-dependent manner, reaching a peak after 1 hr of incubation and beginning to decrease after 3 hr. To confirm the role of Akt in GSK-3β phosphorylation and the subsequent steps of this pathway, we coincubated BEAS-2B cells with chrysotile and a specific Akt inhibitor. After this coincubation, the levels of GSK-3β phosphorylation and SNAIL-1 accumulation in the nucleus were comparable to those in controls. Furthermore, after a long (72 hr) exposure, the Akt inhibitor partially blocked both SNAIL-1 accumulation in the nucleus and the E-cadherin decrease observed after exposure to chrysotile only.

To our knowledge, we are the first to report that chrysotile induces EMT in BEAS-2B cells through a molecular mechanism that appears to involve TGF-β and its intracellular effectors Akt/GSK-3β/SNAIL-1. However, the incomplete recovery of the investigated epithelial and mesenchymal markers and of the intracellular mediators can be explained by considering TGF-β as just one of the possible mediators of the asbestos-induced EMT event in these cells.

## Conclusions

Asbestos is a complex stimulus that elicits a variety of cellular responses through multiple molecular pathways. Thus, several different mechanisms may be activated as a consequence of asbestos exposure. Additional mechanisms may also be identified, but our findings suggest that chrysotile is able to trigger EMT in BEAS-2B cells, at least in part, through a molecular mechanism involving TGF-β and its intracellular effectors Akt/GSK-3β/SNAIL-1. Our findings suggest just one of the possible molecular mechanisms supporting the morphological transformations typical of EMT involved in asbestos-related diseases; therefore, it is conceivable that additional mechanisms will be unveiled in the future.

## Supplemental Material

(502 KB) PDF
