# Supplementary material for: Effects of Chrysotile Exposure in Human Bronchial Epithelial Cells: Insights into the Pathogenic Mechanisms of Asbestos-Related Diseases
Source: Environ Health Perspect. 2015 Dec 18;124(6):776–84. doi: 10.1289/ehp.1409627 (PMC4892914; doi:10.1289/ehp.1409627)
Supplement: (502 KB) PDF [file ehp.1409627.s001.acco.pdf]

Environ Health Perspect
DOI: 10.1289/ehp.1409627

Note to readers with disabilities: *EHP* strives to ensure that all journal content is accessible to all readers. However, some figures and Supplemental Material published in *EHP* articles may not conform to 508 standards due to the complexity of the information being presented. If you need assistance accessing journal content, please contact ehp508@niehs.nih.gov. Our staff will work with you to assess and meet your accessibility needs within 3 working days.

Supplemental Material

Effect of Chrysotile Exposure in Human Bronchial Epithelial Cells: Insights on the Pathogenic Mechanisms of Asbestos-Related Diseases

Giulia Rossana Gulino, Manuela Polimeni, Mauro Prato, Elena Gazzano, Joanna Kopecka, Sebastiano Colombatto, Dario Ghigo, and Elisabetta Aldieri

Table of Contents

Figure S1. Chrysotile asbestos effect on cell morphology and EMT marker protein expression in NuLi-1 cells

Figure S2. Chrysotile asbestos effect on cell morphology and EMT marker protein expression in A549 cells

Figure S3. Effect of chrysotile asbestos on the production of ROS in BEAS-2B cells

Figure S4. Neutralizing TGF-β antibody effect in BEAS-2B cells

Figure S5. Chrysotile asbestos effect on the Smad2-dependent pathway in BEAS-2B cells

Figure S1.

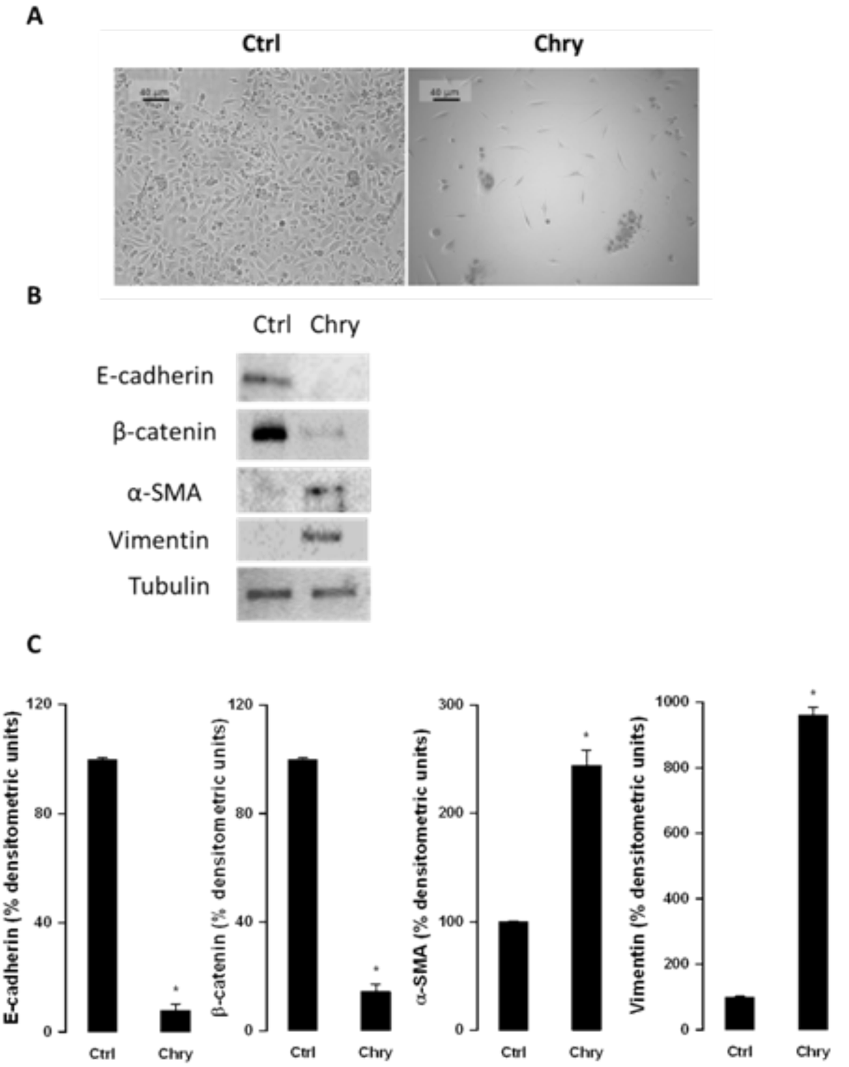

Figure S1. Chrysotile asbestos effect on cell morphology and EMT marker protein expression in NuLi-1 cells. NuLi-1 cells were cultured for 96 h without (Ctrl) or with 1 μg/cm^2 of chrysotile (Chry). **A**) Representative microscope images are shown (10x; scale bar = 40 μm). **B**) Expression of epithelial (E-cadherin, β-catenin) and mesenchymal (α-SMA, vimentin) markers checked by Western blotting. Tubulin was used as loading control. The image is representative of three independent experiments giving similar results. **C**) Densitometry data are presented as the percent decrease or increase in the protein expression levels *vs* respective control. Significance *vs* respective control: *$p<0.0001$.

Environ Health Perspect
DOI: 10.1289/ehp.1409627

Figure S2.

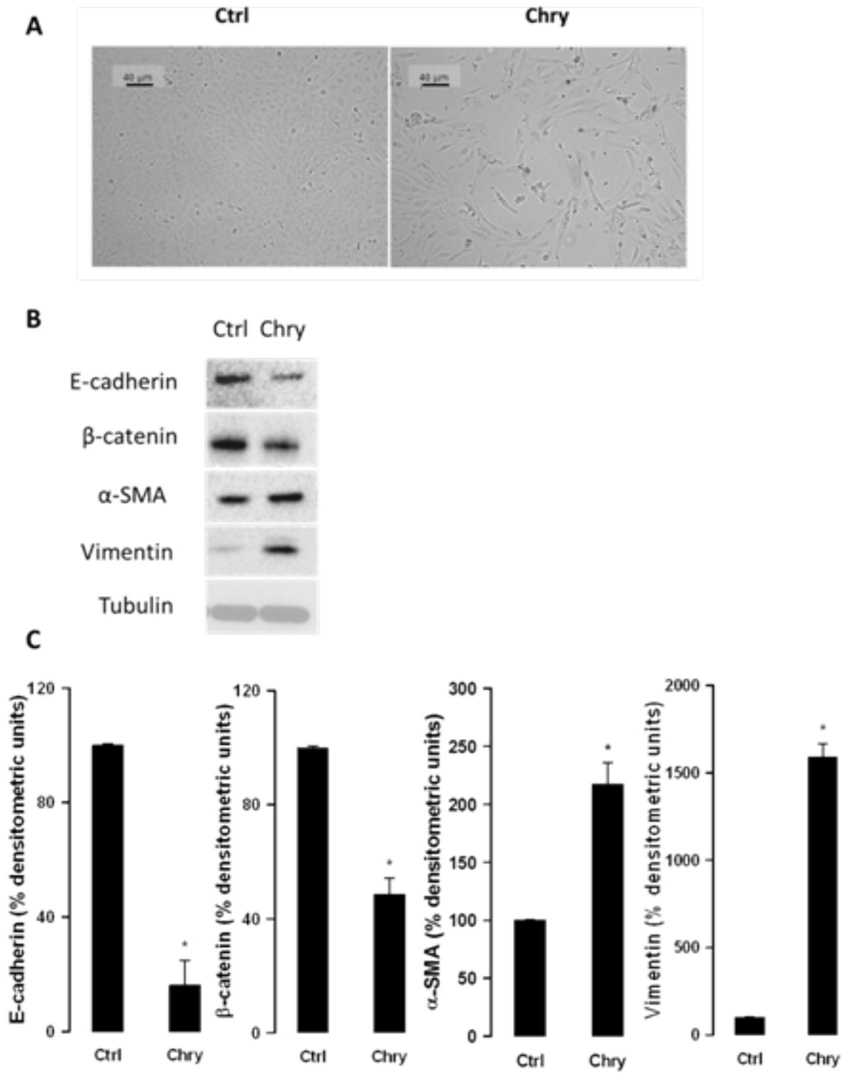

Figure S2. Chrysotile asbestos effect on cell morphology and EMT marker protein expression in A549 cells. A549 cells were cultured for 96 h without (Ctrl) or with 5 μg/cm^2 of chrysotile (Chry). **A**) Representative microscope images are shown (10x; scale bar = 40 μm). **B**) Expression of epithelial (E-cadherin, β-catenin) and mesenchymal (α-SMA, vimentin) markers checked by Western blotting. Tubulin was used as loading control. The image is representative of three independent experiments giving similar results. **C**) Densitometry data are presented as the percent decrease or increase in the protein expression levels *vs* respective control. Significance *vs* respective control: *p<0.0001.

DOI: 10.1289/ehp.1409627

Figure S3.

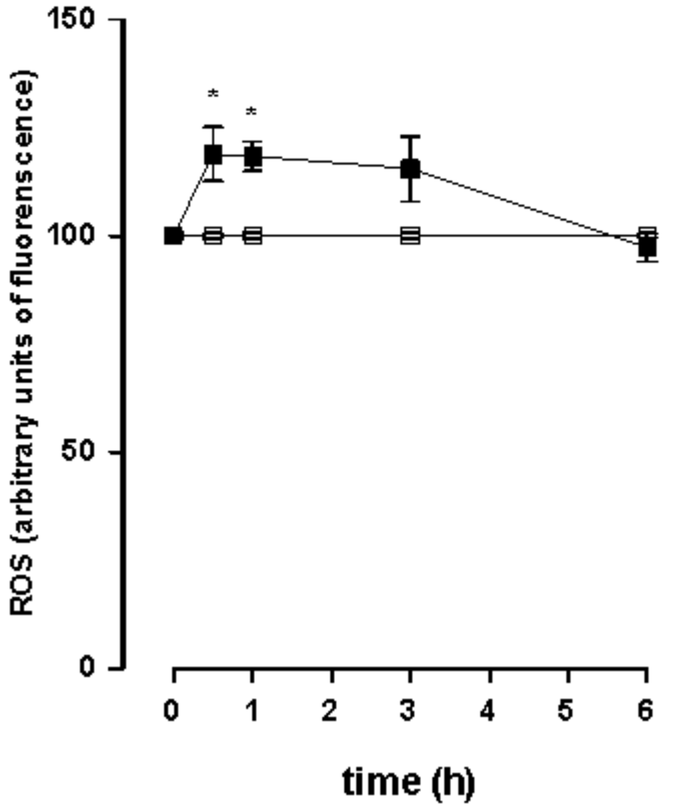

Figure S3. Effect of chrysotile asbestos on the production of ROS in BEAS-2B cells. BEAS-2B cells were incubated for 0, 30 min, 1, 3 and 6 h in the presence (black squares) or absence (white squares) of 1 µg/cm^2 of chrysotile. Cells where then loaded with DCFH-DA and the fluorescence was measured. Each measurement was performed in duplicate and data are presented as mean ± SEM (n = 3). Significance *vs* respective control: *p<0.05.

Figure S4.

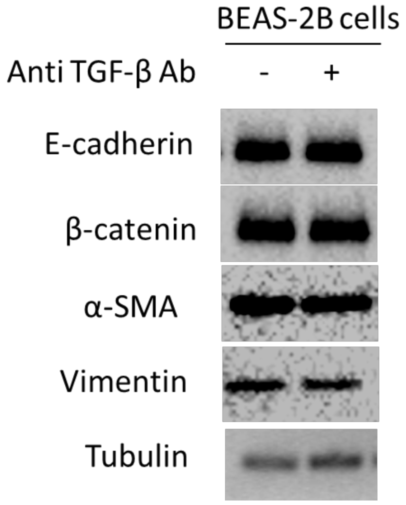

Figure S4. Neutralizing TGF-β antibody effect in BEAS-2B cells. BEAS-2B cells were cultured for 72 h in the presence or absence of 5 ng/ml TGF-β blocking antibody (Anti TGF-β Ab). Expression of epithelial (E-cadherin, β-catenin) and mesenchymal (α-SMA, vimentin) markers was checked by Western blotting. Tubulin was used as loading control. The image is representative of three independent experiments giving similar results.

Figure S5.

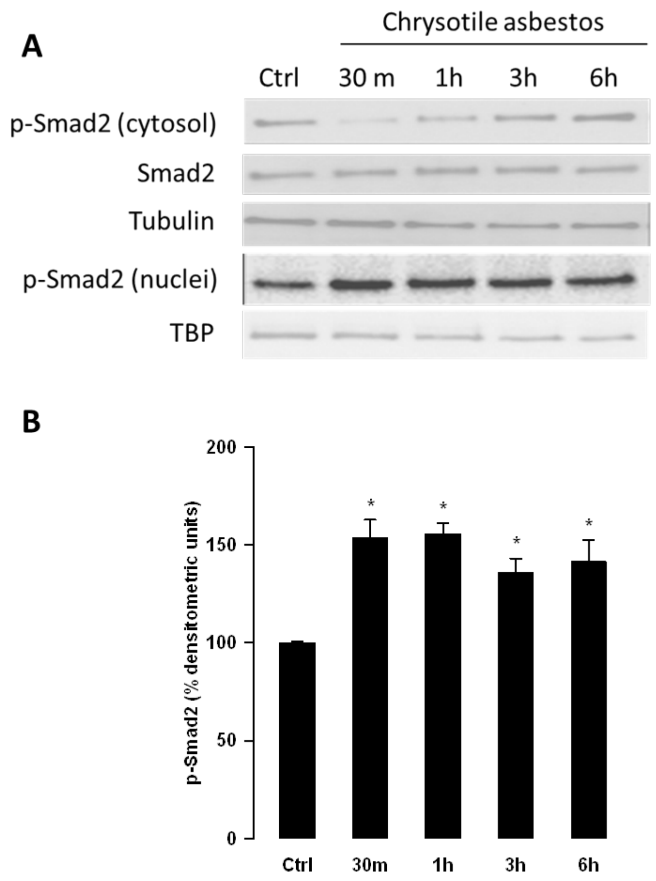

Figure S5. Chrysotile asbestos effect on the Smad2-dependent pathway in BEAS-2B cells. A) BEAS-2B cells were incubated in the absence (Ctrl, 6 h) or presence of 1 μg/cm^2 chrysotile for 30 min (30 m), 1, 3 and 6 h. Smad-2 and p-Smad-2 expression was examined through Western blotting. Tubulin and TBP were used as loading control for cytosolic and nuclear extracts respectively. The image is representative of three independent experiments giving similar results. **B**) Densitometry data of p-Smad2 accumulation in the nuclei of BEAS-2B cells presented as the percent increase *vs* control (Ctrl, 6h). Data are presented as mean $\pm$ SEM (n = 3). Significance *vs* control: *p<0.0001.
